# Application of virtual reality on non-drug behavioral management of short-term dental procedure in children

**DOI:** 10.1186/s13063-021-05540-x

**Published:** 2021-08-23

**Authors:** Longkuan Ran, Nan Zhao, Lin Fan, Pinping Zhou, Chao Zhang, Cong Yu

**Affiliations:** 1 Department of Anesthesiology, Stomatological Hospital of Chongqing Medical University, Chongqing, China; 2 Chongqing Key Laboratory of Oral Diseases and Biomedical Sciences, Chongqing, China; 3 Chongqing Municipal Key Laboratory of Oral Biomedical Engineering of Higher Education, Chongqing, China

**Keywords:** Virtual reality, Dental anxiety, Pain, Short-term dental procedure

## Abstract

**Background:**

Due to the inherent characteristics of immersion, imagination, and interactivity in virtual reality (VR), it might be suitable for non-drug behavior management of children in dental clinics. The purpose of this trial was to measure the role of VR distraction on behavior management in short-term dental procedures in children.

**Methods:**

A randomized clinical trial design was carried out on 120 children aged between 4 and 8 years to identify the comparative efficacy of VR and tell-show-do (TSD) to improve behavioral management during dental procedures. The primary outcomes were evaluated anxiety, pain, and compliance scores in perioperative children. The levels of operative anxiety and pain were assessed using the Children’s Fear Survey Schedule-Dental Subscale (CFSS-DS) and Wong Baker FACES Pain Rating Scale (WBFS), respectively. The Frankl Behavior Rating Scale (FBRS) was tested before and during dental procedures. The length of the dental procedure was compared between both groups after treatment.

**Results:**

The average anxiety and behavioral scores of the VR group significantly reduced compared with the control. The decreased anxiety score for the VR group and control group were 8 (7, 11) and 5 (5, 7), *p* < 0.05. The compliance scores of the control group during treatment were 3 (2, 3), and the same in the VR intervention were 3 (3, 4), *p* = 0.02. A significant reduction in pain was observed when using VR distraction (*p* < 0.05). Comparing the length of the dental procedure, the VR group (19.0 2 ± 5.32 min) had a shorter treatment time than the control group (27.80 ± 10.40 min).

**Conclusion:**

The use of VR significantly reduced the anxiety and pain of children and the length of the dental procedure and improved the compliance of children that underwent short-term dental procedures without an adverse reaction.

**Trial registration:**

Chinese Clinical Trial Registry, ChiCTR2000029802. Registered on February 14, 2020

## Introduction

Successful behavioral management might be related to the following two variables: anxiety, which is a psychological state and can be modified and controlled with psychological techniques, and pain, which is an unpleasant sensory and emotional experience [1]. For children’s cognition levels during a dental procedure, it is necessary to conduct behavioral management; therefore, the children can cooperate with the pediadontist to complete the treatment.

In Southwest China, a substantial proportion of children that require dental care are unable to collaborate well with doctors and nurses due to dental anxiety (DA). The increase in pain, tension, and fear-related behaviors during dental procedures was defined as DA; it can be expressed as rapid heart rate, muscle tension, and even syncope [2]. DA refers to a universal level of stress that is characteristic of an individual and might have a constant level during the life span. The emotion interferes significantly with personal daily life, career development, or relationships. Various studies found that the incidence of DA was 20–43%, which depended on the age of the child. In a recent survey, DA produced the majority of clinical problems in pediatric dental treatment [3].

For the treatment of DA, drugs were generally used in the Department of Anesthesiology of Stomatological Hospital affiliated to Chongqing Medical University (Yubei District, Chongqing, China), such as nitrous oxide (N_2_O) and sevoflurane. The N_2_O sedation can only be applied for patients that score three or four on the Frankl Behavior Rating Scale (FBRS) [4, 5]. Because the children are awake and have no other way to distract their attention, many children do not cooperate with pedodontists during a dental procedure. The child’s parents might be concerned about possible damage from sevoflurane inhalation anesthesia, because the children and their families need to carry out general anesthesia-related preoperative preparation: waiting for the appointment, fasting and drinking, risk of apnea, and recovery after anesthesia. Therefore, some children did not receive timely treatment, which results in lifelong poor oral health, and can cause long-term psychological trauma [6]. Therefore, timely and effective management of DA is central to improving the mental and physical health of children with oral diseases.

Virtual reality technology (VR) creates a highly realistic virtual, three-dimensional (3D) environment that provides various sense stimulate (e.g., sense of vision, sense of hearing, touch, and sense of smell) for the user to escape the real world [7, 8]. By stimulating visual, auditory, and proprioceptive sensations, VR acts as a distraction to interfere with the patient’s handling of noxious stimuli [8]. In the last decades, VR has been applied to different healthcare settings. In particular, VR has reported in many clinical trials, such as trauma rehabilitation [9, 10], are of burns [11, 12], cancer treatment [13], operation training [14], and weight-related disorders [15]. Sato and Sarig-Bahat performed VR on complex regional pain syndromes [16] and chronic neck pain [17].

The analgesic effects of VR distraction reduce negative emotions (i.e., anxiety) and lead to positive emotions [18]. In some studies, VR distraction has been used to relieve pain and anxiety [7, 8, 11, 19–23]. Similarly, VR has been used as a distraction intervention to relieve pain during the perioperative period in dental surgery [19, 23]. The application of VR technology in the behavioral management of children’s dental procedures is limited. In addition, recent reports have stated that it reduces pain and anxiety in the dental setting and procedures [24, 25], but only for single dental procedures and the suitable duration of the treatment involved needs to be explored. In this study, the role of VR in the non-drug behavioral management of children is measured with short-term and simple dental procedures.

## Methods

### Setting and patients

This randomized clinical trial recruited 120 preschoolers aged 4–8 years who came to the Stomatological Hospital of Chongqing Medical University for dental treatment. This study followed the Declaration of Helsinki on medical protocol and ethics and the Regional Ethical Review Board of the Stomatological Hospital of Chongqing Medical University approved the trial.

### Inclusion criteria

Consenting children (aged 4–8 years, ASA I-II) with a Children’s Fear Survey Schedule-Dental Subscale (CFSS-DS) questionnaire > 19 [26]. The time of the dental procedure (caries treatment, extraction of deciduous teeth, incision of abscess, and root canal therapy) was expected to be < 30 min.

### Exclusion criteria

The exclusion criteria were children or their families that could not agree, and their families were concerned that VR could have an impact on the eyes of the child and for other reasons that could interfere with wearing the VR glasses, such as those that required glasses for myopia. Since VR might cause motion sickness in some users, children were excluded with a history of motor diseases, motor nausea, or vomiting. Children with a history of epileptic or epileptic seizures were excluded, because there are some reports that VR has a theoretical risk of inducing seizures. Unpleasant treatment experience increases anxiety and pain during the following dental sessions, which results in increased pain perception. Therefore, in this study, subjects were excluded if they had a previous serious dental experience [27].

**Fig. 1:**
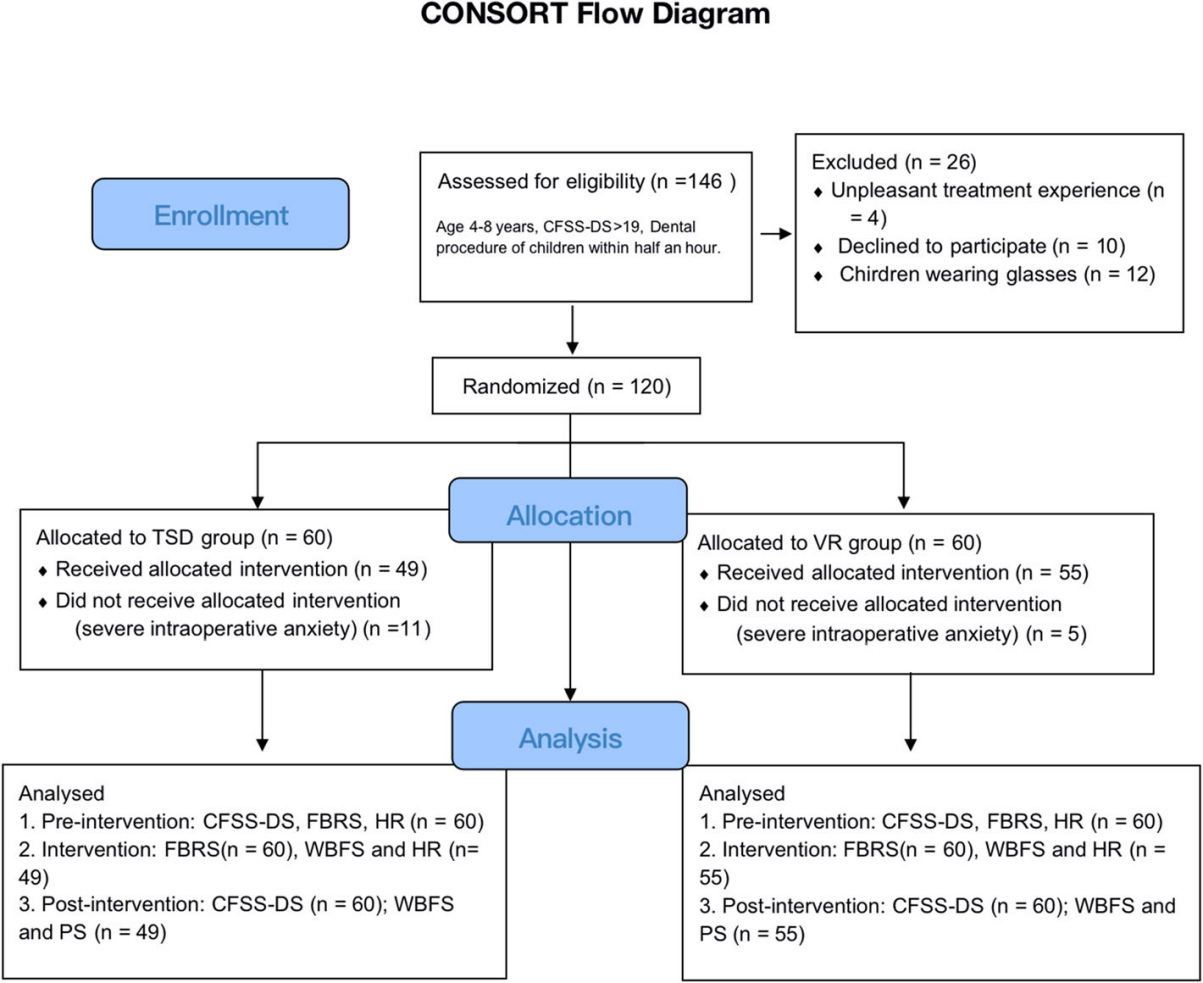
CONSORT flow diagram for the randomized trial. CFSS-DS, Children’s Fear Survey Schedule-Dental Subscale; TSD, tell-show-do; VR, virtual reality; FBRS, Frankl Behavior Rating Scale; WBFS, Wong Baker FACES Pain Rating Scale; HR, heart rate; PS, patient satisfaction

If a child had a serious fear or severe movement during the intervention, the trial was terminated immediately. Figure 1 shows the CONSORT Flow Diagram for the trial.

### Technical specifications

HTC (Hsinchu, Taiwan, China)’s VIVE VR helmet was used, which is commercial, widely used, with a short delay time for video scenes, and is not prone to head vertigo. The VIVE is composed of 32 sensors for 360° motion tracking, two 2160 × 1200 combined resolution AMOLED screens, and a 90-Hz refresh rate. The helmet was connected to an ASUS Game notebook with an Intel Core i7-8820K processor, 16-GB RAM, and an NVIDIA GeForce GTX 1070 graphics card. The virtual environment allowed the user to navigate naturally, which was created through a 110° field of view for immersion.

### Procedure

**Fig. 2:**
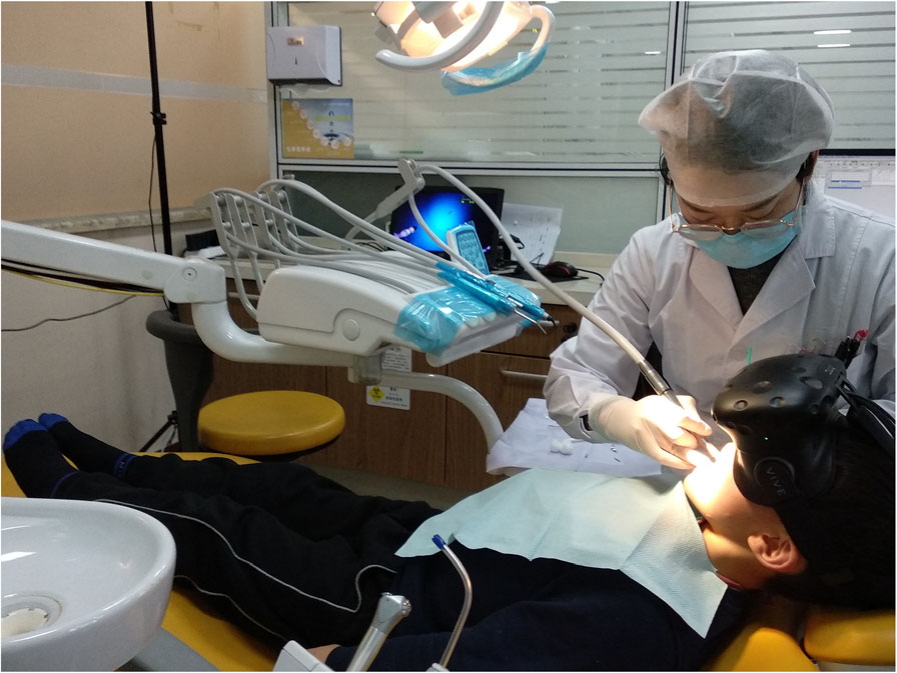
A scene in which HTC’s VIVE VR helmet is being used during a dental procedure

Patients were randomly allocated to two conditions using randomization software (STATA software version 15.1). Eligible children’s parents or caregivers were informed about the trial by the anesthetists, and informed consent was obtained preoperatively. When they had agreed to participate in the study, personal medical data were collected by researchers and the baseline anxiety was assessed by CFSS-DS (T0 = time after signing the consent). Then, the anesthetist nurse randomly allocated children to the VR intervention or to the control group (children only received tell-show-do (TSD) as usual). Block randomization was performed based on the type of dental procedure: caries treatment, extraction of deciduous teeth, incision of abscess, and root canal therapy. After randomization, the VR intervention took place in a separate room under the guidance of the nurse anesthetist, and children in the TSD group were admitted to the other room. Both groups were treated by experienced pediatric dentists. Figure 2 shows an example of the dental procedure using HTC’s VIVE helmet.

The assessment per time point was performed. FBRS was scored before intervention (T1 = 5 min before dental procedure) and remeasured at the moment of local anesthetic injection (T2). CFSS-DS and patient satisfaction (PS) scores were performed 5 m after the end of treatment (T3). Because VR intervention could cause adverse events, dizziness, nausea, vomiting, and epilepsy, they were followed-up during treatment. In addition, changes in heart rate and peripheral capillary oxygen saturation between both groups before, during, and after dental treatment were measured.

### VR intervention

**Fig. 3:**
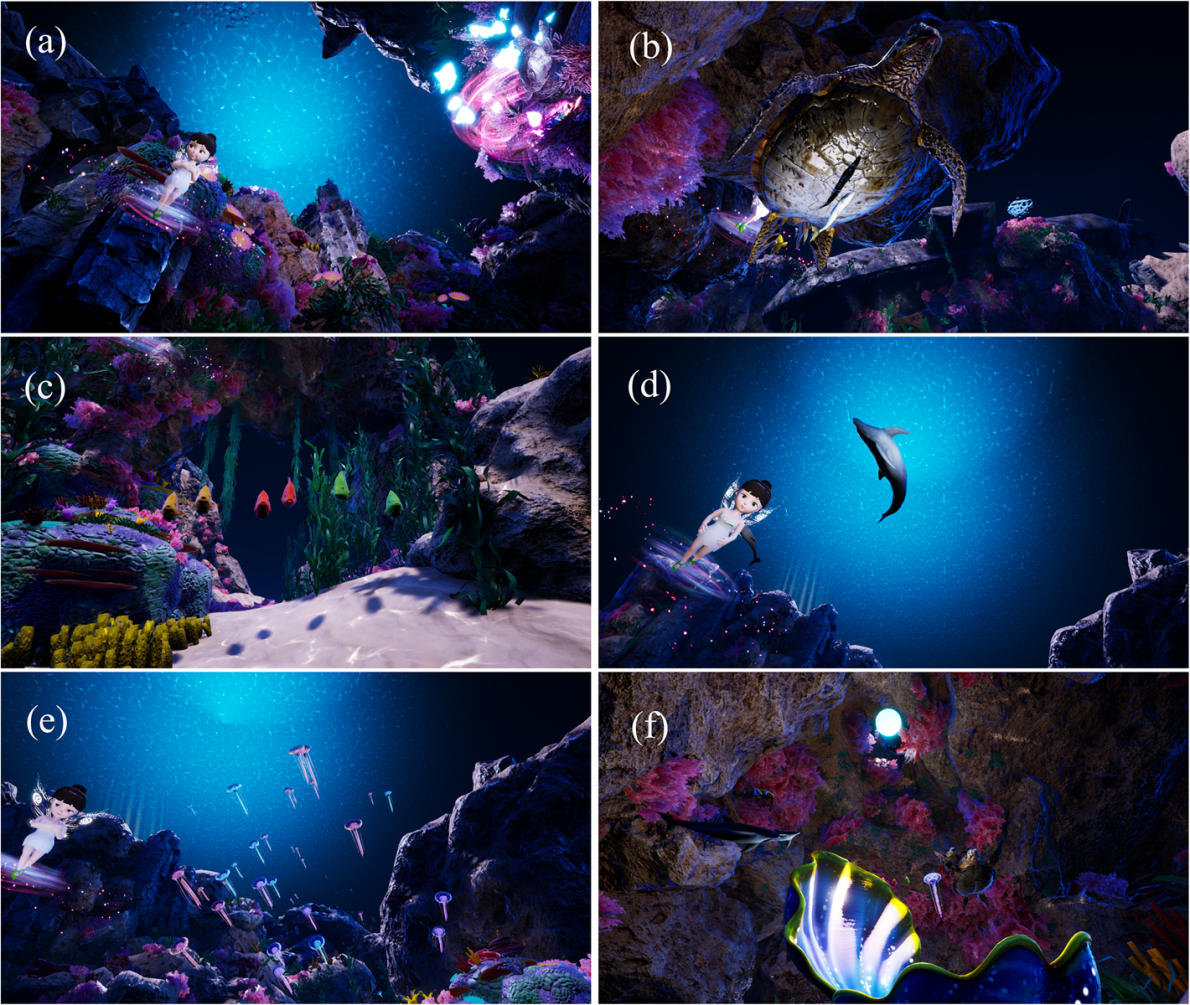
Screenshot of custom scenario

Before the dental procedure, the patient was shown the corresponding scenes, specific inducers, and background music that was developed with psychologists, which could attract their attention and relax them. The children lay down on the dental chair and did not shake their heads left and right, which caused the treatment to be interrupted. The nurse anesthetist put the helmet and earphones on the children, who entered a virtual world where they could follow a set route and watch different information expressed in the scenes. The story began in a shallow sea world with a soft environment. The undersea world could only be saved when the undersea creatures shared their most precious things to nourish a rare pearl. First, “I” am in a shell, and a little elf introduces the creatures of the sea and their precious spirits. Second, a sea anemone protected the clown fish when the clown fish reduces the surface material of the anemone, and they shared this precious friendship by helping each other (Fig. 3a). Then, the children were introduced to the ancient precious and tenacious vitality of animals: turtles (Fig. 3b), parrot fish solidarity (Fig. 3c), and the dolphin’s helpful spirit (Fig. 3d). Scenes were switched for the children to introduce a beautiful and dangerous jellyfish, which shared its valuable ability to predict storms (Fig. 3e). Finally, the pearl was born from the precious spiritual nourishment from thousands of marine creatures and the undersea world was restored to its former peace and tranquility (Fig. 3f). In addition, the virtual environment was displayed on the ASUS notebook, and therefore, the accompanying families could see what the child was viewing.

### Primary outcomes

The primary outcomes were CFSS-DS, WBFS, and FBRS in the VR group, compared with those in the TSD group. The CFSS-DS was monitored at T0 and T3, while FBRS was evaluated at T2 and T3, and WBFS (score was from 0 to 10; 0 = no pain, 10 = extreme pain) was recorded at T2 and T3 points.

### Statistical analyses

Data were analyzed using SPSS software version 22 (IBM, Chicago, USA). The statistically significant difference was set at *p* < 0.05. A Chi-squared test was used to assess gender difference, ASA physical status, type of dental procedure, and local anesthetic between both groups. All quantitative variables were presented as mean ± standard deviation, and the analysis of variance or a nonparametric test was performed for comparisons according to the data distribution. Mann-Whitney *U* tests were used to compare the downward trend for the level of anxiety and FBRS.

## Results

The trial was composed of 25 girls and 35 boys in the VR group and 32 girls and 28 boys in the TSD group, respectively. The mean ages in the VR and TSD groups were 5.59 ± 0.92 and 5.66 ± 0.99 years, *p* = 0.69. Similarly, there were no significant differences between both groups in the means for gender, ASA physical status, type of dental procedure, and location and type of dental procedure (Pearson’s Chi-squared test, *p* > 0.05; Table 1).

**Table 1:** Patient and dental procedures

	VR^a^ (***n*** = 60, %)	TSD^b^ (***n*** = 60, %)	***p-***value
**Age (years)**	5.59 ± 0.92	5.66 ± 0.99	0.69
**Gender**			
Male	35 (58.33)	28 (46.67)	0.20
Female	25 (41.67)	32 (53.33)	
**ASA**^c^**physical status**			
I	43 (71.67)	46 (76.67)	0.53
II	17 (28.33)	14 (23.33)	
**Dental procedure**			
Caries treatment	27 (45.00)	20 (33.33)	0.63
Extraction of deciduous teeth	12 (20.00)	15 (25.00)	
Incision of abscess	11 (18.33)	13 (21.67)	
Root canal therapy	10 (16.67)	12 (20.00)	
**Local anesthetic**			
Primacaine	48 (80.00)	46 (76.67 )	0.39
Not used	12 (20.00)	14 (23.33)	

^a^*VR* virtual reality group

^b^*TSD* tell-show-do group

^c^*ASA* American Society of Anesthesiologists

*p-*value < 0.05 statistically significant

The mean anxiety scores decreased significantly after VR distraction. In the VR group, the CFSS-DS was 34.17 ± 5.81 before the intervention, which decreased to 24.77 ± 6.98 after VR distraction. In the TSD group, the anxiety scores were 34.08 ± 8.42 and 27.98 ± 7.41, respectively (*n* = 60). The anxiety score between VR intervention and TSD were statistically different after a dental procedure (Table 2). Table 2 shows the downward trend of variations in anxiety scores between both groups and the differences were statistically significant.

**Table 2:** Anxiety, pain, PS, and time of dental procedure in both groups

	VR^a^ (***n*** = 60)	TSD^b^ (***n*** = 60)	***p-***value	VR (***n*** = 55)	TSD (***n*** = 49)	***p-***value
**Anxiety**						
CFSS-DS^c^						
Pre-intervention (T0)	34.17 ± 5.81	34.08 ± 8.42	0.95	33.15 ± 4.82	31.45 ± 5.97	0.11
Post-intervention (T3)	24.77 ± 6.98	27.98 ± 7.41	0.02*	23.34 ± 5.23	25.43 ± 5.20	< 0.05*
Downtrend (T0-T3)	8 (7, 11)	5 (5, 7)	< 0.001*	8 (7, 12)	5 (5, 7)	< 0.001*
FBRS^d^						
T1	2 (2, 3)	2 (2, 3)	0.26	2 (2, 3)	2 (2, 3)	0.12
T2	3 (3, 4)	3 (2, 3)	0.02*	3 (3, 4)	3 (3, 3)	0.11
**Pain**						
WBFS^e^						
T2 (observed)				1.58 ± 1.08	2.86 ± 0.96	< 0.001*
T3 (observed)				1.62 ± 1.13	3.59 ± 1.19	< 0.001*
**PS**^f^**(T3, score 0–100)**				88.33 ± 7.15	76.78 ± 8.49	< 0.001*
**Length of dental procedure (m)**						
T1 to T3				19.02 ± 5.32	27.80 ± 10.40	< 0.001*
Caries treatment				19 (16, 22)	30 (25, 30)	< 0.001*
Extraction of deciduous teeth				14.75 ± 2.77	19.93 ± 9.01	0.07
Incision of abscess				22.40 ± 3.89	26.09 ± 6.80	0.15
Root canal therapy				23.57 ± 4.39	42.43 ± 8.40	< 0.001*

^a^*VR* virtual reality group

^b^*TSD* tell-show-do group

^c^*CFSS-DS* Children’s Fear Survey Schedule-Dental Subscale

^d^*FBRS* Frankl Behavior Rating Scale

^e^*WBFS* Wong Baker FACES Pain Rating Scale

^f^*PS* Patient satisfaction

*T0*, signature of informed consent statement; *T1*, before intervention; *T2*, the moment of local anesthetic injection; *T3*, the end of treatment

*p-value < 0.05 statistically significant

The Mann-Whitney *U* tests of FBRS for the VR group in the pre-intervention were 2 (2, 3). Similarly, the T2 of VR distraction treatment were 3 (3, 4), which represented that VR distraction improved patient compliance. In the control group, the FBRS before intervention and the time of the maximum procedure pain were 2 (2, 3) and 3 (2, 3), respectively. This indicated that VR increased compliance (*p* = 0.02; Table 2).

Five children (8.33%) in the VR group had severe intraoperative anxiety and stopped treatment in fear, compared with 11 cases (18.33%) in the TSD group (*p* < 0.05). The CFSS-DS score of the children after the exclusion of the previous patient found that VR interference relative to the control group could significantly alleviate the anxiety of the children, *p* < 0.05. The FBRS of both groups at T2 had no statistical difference.

During the operation, the pain score of the VR group (1.58 ± 1.08) was lower than that of the control group (2.86 ± 0.96). In addition, the results showed that the VR intervention at the end of treatment, related to the control of operation pain, worked best (*p* < 0.001) in the patients. Comparing the length of the dental procedure, the VR group (19.02 ± 5.32 min) had a shorter treatment time than the control group (27.80 ± 10.40 min). The results of this trial indicated the decreased treatment time was particularly significant in carie treatment and root canal therapy and was statistically significant (*p* < 0.001). Table 2 shows that the overall PS of the dental procedure with VR intervention (88.33 ± 7.15) was significantly higher than the TSD group (76.78 ± 8.49).

**Fig. 4:**
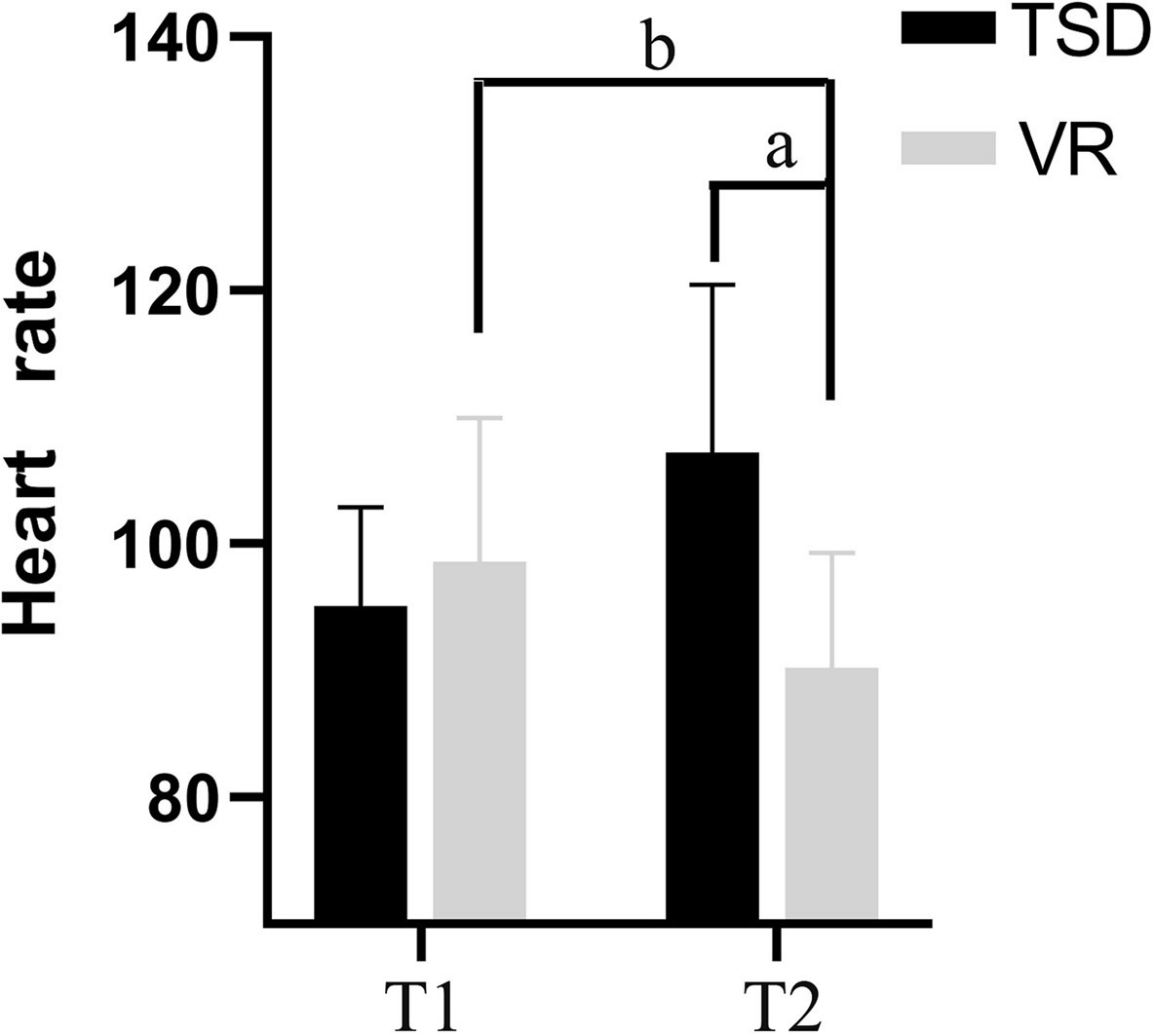
Changes in heart rate before and during the dental treatment between both groups. VR, virtual reality group; TSD, tell-show-do group; T1, 5 min before dental procedure; T2, the moment of local anesthetic injection. ^a,b^*p*-value < 0.05

After monitoring the physiological signs, the VR group had decreased heart rates and the control group had the opposite (*p <* 0.05; Fig. 4). There was no significant difference in the SPO_2_, which was detected before and after the intervention between both groups.

## Discussion

Our group has been engaged in oral-related sedation and analgesia for a long time; therefore, we considered whether new methods could be used to relieve the DA in this group of children [28–30]. Therefore, this trial focused on the effectiveness of compliance changes during VR distraction in 4–8-year-old children during a short invasive dental treatment [6]. The results of this trial supported that VR was associated with a larger decrease in behavioral avoidance compared with TDS intervention. The anxiety score of both groups before and after the intervention was monitored, and the VR group decreased more than the control group; however, the compliance score of the children increased. Aminabadi et al. reported that VR eyeglasses successfully decreased pain perception and the anxiety state during dental treatment in 4–6-year-old children [31]. Similarly, Shetty observed that VR distraction could be used as a successful behavior modification method in 5–8-year-old children that underwent short invasive dental treatments [32]. Therefore, this trial and references supported that immersion in a virtual environment could help to control DA, improve compliance, and relieve pain during pediatric dental treatment in children [31, 32]. Researchers showed that the effects of VR techniques on pain perception were beyond simple distraction [33]. In addition, by diverting attention from an unpleasant environment setting to a pleasant and absorbing virtual world, VR diminished the patient’s physical pain experience [10, 34, 35]. By relieving anxiety and reducing pain, children cooperated better with the treatments, which included caries treatment and root canal therapy.

In addition, the treatment time for the VR group was significantly shorter than the control group. In a previous clinical treatment, general anesthesia by inhaling sevoflurane was an invasive medical method that has been used on most children, who had difficulty in adapting to TSD behavioral induction. Some children might have received delayed treatment because of family concerns about general anesthesia or their lack of cooperation with behavioral induction, which resulted in poor prognosis and long-term tooth problems. This trial suggested that the customized VR content applied in this study allowed children to be treated as quickly as possible and might reduce the frequency of patients seeking medical treatment and reduce the number of outpatients. In China, the ratio of health workers to people is significantly lower than the global average, especially pediatricians [36]. This could be beneficial for doctors and patients.

This trial has several limitations. First, the scales for anxiety and pain in children were relatively single, different clinometric tools, and assessments were not included. Second, other factors that affect children’s behavior were not accounted for. Some researchers reported that several factors, such as age, gender, type of dental treatment, parental anxiety, and socioeconomic status were associated with anxiety should be assessed, because these might influence the efficacy of VR [37]. The evidence reported that older children considered the VR technique as a very simple game, and they had a have a lower level of distraction [33]. This study identified five children who were uncomfortable with VR distraction and terminated the trial, considering that some of them were possible terminated for these reasons. But the average age analysis of the five children was not statistically significant. In addition, their anxiety scores were > 35 before and after treatment. Third, the pedodontists and the patients were not blinded to the interventions due to the apparent difference between both groups. During future work, the deficiency could be overcome by watching different VR animations in different groups. Fourth, the VR in this trial only had one animation content, which might be considered a limitation, because the stimuli that trigger DA might differ for each individual. Based on the current data that uses VR, anxiety score, and mobile internet APP to collect family members’ awareness and anxiety about dental diseases, to achieve fewer generic intervention scenarios the next step could be hierarchical customization of VR content. In addition, VR should improve and its use increased in the children’s department of stomatology. Finally, a set of intelligent biofeedback mechanisms should be formed to achieve closed-loop control of anxiety and pain in children; therefore, children will receive rapid, timely, and comfortable oral treatment.

This trial focused on the effectiveness of compliance changes in VR distraction in 4–8-year-old children during short-term invasive dental procedures. The VR distraction used nonintrusive methods; therefore, the children’s parents or caregivers have not been concerned that general anesthesia might affect intelligence and learning ability. The adverse effects on vision and hearing have not been reported in this study and literature [31, 38–40]. Even with repeated dental treatments, parents and children are much more receptive to other methods.

## Data Availability

The ethical approval does not permit the sharing of the entire data that we have acquired, but the information required is already provided in the main manuscript.
